# Recent physician strike in Israel: a health system under stress?

**DOI:** 10.1186/2045-4015-2-33

**Published:** 2013-08-15

**Authors:** Leonora G Weil, Gabi Bin Nun, Martin McKee

**Affiliations:** 1 London School of Hygiene and Tropical Medicine, 5-17 Tavistock Place, London, WC1H 9SH, UK; 2 Ben Gurion University of the Negev, Beer Sheva, Israel

**Keywords:** Physician, Doctor, Strikes, Health systems, Israel

## Abstract

In 2011, a series of physician strikes in Israel followed eight months of unsuccessful negotiations with the government (Ministry of Health and the Ministry of Finance). Strikes by physicians may be a warning that all is not well in a health system and protestors have claimed that they signify a system failure. In contrast, others argue that strikes have been a feature of the Israeli health system from its inception and should not be a cause for alarm. This paper analyses the Israeli health system from the perspective of the strikers' demands using the World Health Organisation’s six health system building blocks as a framework, including: service delivery; health workforce; information; medical products, vaccines and technologies; leadership and governance; and financing. While we recognise that the immediate causes of the 2011 strikes were concerns about salaries and working conditions, we argue that a complex set of interacting factors underlie the strikers' demands, resonating with issues relating to five of the WHO building blocks. We argue that of the five, three are most significant and limit progress with all the others: a disgruntled health workforce, many of whom believe that striking is the only way to be heard; a lack of leadership by the government in understanding and responding to physicians' concerns; and a purported information insufficiency, manifest as a lack of critique and analysis that may have prevented those at the top from making a reliable diagnosis of the system’s problems. This paper argues that there are cracks within the Israeli health system but that these are not irresolvable. The Israeli health system is a relatively new and popular health system, but there are no grounds for complacency.

## Introduction

Between April and November in 2011 a series of partial strikes were initiated by Israeli physicians, mainly involving those working in hospitals. These strikes, the longest running in Israel’s history [1], followed eight months of unsuccessful negotiations with the government (Ministry of Health and the Ministry of Finance).

In brief, the Israel Medical Association (IMA) - the professional association of Israeli physicians - demanded increased hospital capacity, employment of an extra 1,000 doctors, that salaries be doubled, that those working in remote areas and in unpopular specialties receive higher pay, and that doctors’ hours be reduced. Doctors also demanded that an increased share of their wages be used as a basis for their employers' contributions to their pensions and that there should be an increased time allocation for each health clinic consultation, from 10 to 12–15 minutes [2-4]. In the background to the strike there was also a demand to allow private practice in public hospitals. This was a partial, rather than a full strike as not all doctors were involved and many of those involved continued to work part of the time. A deal, agreed with the Ministry of Finance in August 2011, fulfilled part of the IMA’s requests but there was a second wave of resignations and rallies in August-November, with protestors accusing the IMA of having ‘sold out’ [4,5].

Physician strikes in Israel are not new and there have been many in its short history, in 1973, 1976, 1983, 1987, 1988–1990 (intermittent), 1999 and 2000 [6]; we have summarised the main features of these in Table 1. The new strike was a departure from the recent past. A 10-year agreement reached in 2000 ushered in over a decade of industrial peace but, once it expired, industrial action soon ensued. So what has happened to fracture this peace? Are these recent strikes simply a manifestation of a disagreement over what constitutes a fair and appropriate level of income for doctors? Are strikes now part of established Israeli medical culture? Or do they represent something deeper; a symptom of emerging cracks within the health system?

**Table 1:** **Summary of features of the main physician strikes in Israel from1976**-**2000**

**Strike**	**Strike duration**	**Actions taken**	**Most important proximate cause of the strike**	**The main outcomes**
1976	58 days [7]	• Closure of hospital outpatient clinics	• On-call payments to physicians	• 2.5% Salary increase
				• Revision of salary supplements
		• Only urgent surgical procedures	• Time off after being on-duty	• Revision of on-duty and on-call payments
		• No patient discharges	• Full implementation of the previous physician agreement	• A change in the promotion system and shortening of the promotion period
			• A study fund for physicians	
			• Opposition to moves to reduce physician numbers	
1983	117 Days [7]	• 90% of doctors on strike [8]	• Additional physician posts	• A payment mechanism for working overtime [7]
			• Doubling of salaries	
		• Most hospitals operated on a “weekend basis” over a 4 month period [6]	• Restriction both of working hours and consecutive hours worked [7]	• Supplemental payment to doctors for hospital work [7]
				• Supplemental payment to interns: 10% of a doctors salary [7]
		• Ended with a hunger strike and mass hospital exodus [6]		• ‘Many believe that the strike also damaged public trust in the physicians and their representatives’. ([6] p66)
1994	I day [7]	• 24 hour ‘warming strike’ by 12,000 doctors including those from public sector hospitals, health centres and community health fund clinics [9]	• Increased doctors salaries [7]	• New promotion grades
				• Increased salary supplements
				• Determining a payment rate for on-calls
			• Increased numbers of doctors making it difficult to find work [9]	• Days off after on calls and study leave
				• A new system for further medical studies
		• Many elective operations and outpatient appointments were cancelled [9]		• A professional advancement mechanism
				• Recognition of the physician as a top specialist [7]
		• Only emergency services were operating [9]		
2000	217 days [7]	• ‘General strikes, disruptions and various sanctions’. [7]	• Salary improvement [6,7]	• A 13.2% salary increase for doctors [7]
			• A remuneration mechanism for further study and absences [7]	• Limitation on consecutive hours that interns and residents work
			• Limits on consecutive hours worked [6]	• Increase in the fixed salary portion of earnings from 35% to 50% [7]
			• The right to private practice in public hospitals [6]	• Extension of the physician pension coverage [7]
				• Study leave entitlement [7]
			• Recognition of out-of hours rotations and on-call duty as part of base pay calculations [6]	• Establishment of a public commission to examine the public health system and physicians’ status [6,7]
			• Higher funding and strengthening of the public health system [6]	• Agreement by the IMA not to strike for a decade [6]
				• Both sides agreed to arbitration for unresolved issues [6]

Sources: Heath System Review, the IMA position papers, the Journal of Medical Ethics, the British Medical Journal [6-9].

This paper examines the last of these questions but, to do so, it must first ask why the strikes occurred, describe the functions of a health system, evaluate how the Israeli system undertakes these functions, and whether it has succeeded or failed in doing so with particular reference to the complaints of those striking.

## Methods

The paper uses mixed methods, with a narrative review of the limited material published including academic and professional literature, media coverage and websites (such as that developed by the Israeli Medical Association) supplemented by interviews with key informants. The search of electronic databases for the review of the academic literature used the MeSH terms ‘Israel’, ‘Strikes, Employee’ and ‘Physicians’ with exploding of the term ‘Physicians’ in MEDLINE and Embase subject headings ‘Israel’, ’trade union’ and exploding of the term ‘physician’. A key word search was used in Web of Science: ‘Israel* AND (strike* or ‘industrial action’) AND (physician* or doctor* or general practi*)’. These yielded 23 results on MEDLINE, 12 results in Embase and 25 results in Web of Science on April 5th, 2013. Most of these articles focussed on the morality of striking and consequences for health outcomes rather than an in-depth examination of their causes. Despite a thorough search, no related academic papers written in Hebrew were identified.

Informants were selected purposively to include hospital management, practicing clinicians, the Israeli Medical Association, academics researching health policy, and the mass media. Eight formal interviews were conducted. The formal interviews were held between April 2012-October 2012 and subjects included the director of a large medical centre, a former deputy director a general hospital and senior official in the Ministry of Health’s Medical Division, a senior public health academic, for a senior official at the Israel Medical Association, a dean of a medical school, a leading health researcher at a think tank, and a leading health journalist. None of those interviewed were directly involved with the negotiations.

An interview guide was used, with ten opening questions based on the World Health Organisation’s (WHO) definition of a health system as *‘all organisations, people and actions whose primary intent is to promote, restore or maintain health’*[10]. It used the WHO’s six building blocks; service delivery; health workforce; information; medical products, vaccines and technologies; financing; and leadership and governance [10]. The roles of the Israeli government and the medical profession were explored with respect to the strikers’ complaints as they relate to these six building block functions. Subsequent lines of questioning developed according to emerging themes. It should be noted that we were not seeking consensus on the rights or wrongs of the strikes but rather to obtain information on the underlying problems in the health system. The interviews were used to fill in gaps not addressed adequately from the other sources and we achieved saturation, suggesting that additional interviews were unlikely to provide additional information.

### The Israeli health system

The Israeli healthcare system has provided universal coverage since the 1995 National Health Insurance Law (NHIL) made it a requirement for all Israelis to join one of four not-for-profit health plans (Sick Funds Clalit, Maccabi, Leumit and Meuchedet) [6,11]. People can move between these health plans. Each year the government determines the benefit package that the health plans must provide. There is some variation across regions and ethnic groups in terms of access and quality of Israel’s health system, as is found in most health systems, and it is recognised that these need to be addressed. The state, through the Ministry of Health, is ultimately responsible for health policy and regulates the health system. The state also provides services not included in the insurance schemes, such as maternal and child care, and owns about 50% of acute hospital beds, thereby acting as a direct service provider [6,11].

#### Privately financed health care

Since 1997, there has been a substantial increase in the share of healthcare that is financed privately [1]. Chernicovsky and Regev [1] report that ‘the share of public expenditure in the total national health expenditure has declined to the lowest level exhibited by those developed countries that provide their residents with universal health insurance – less than 60 percent in 2010’.

According to the Household Expenditure Survey [12] the private financing, which accounts for roughly 40% of total financing, is itself composed of the following: private health Insurance (32%), dental care (26%), medications (15%) and other (27%). As Chernichovsky and Navon [13] note, the various components, and sub-components, of private health expenditure vary substantially in the extent to which they address basic needs which Israeli society might consider financing publicly.

#### Physicians’ remuneration

Physicians pay is determined by the setting in which they work, summarised in Table 2. In part due to definitional problems, but also the informal nature of some employment, as would apply equally to all countries where the state is not a monopoly employer, the percentage of doctors working privately is not known. Officially, doctors can practice privately only in private hospitals or in the Jerusalem voluntary hospitals, and not in public hospitals. There are, however, doctors who, in return for under the table payments will practice privately in public hospitals [6]. The extent of this practice is unknown.

**Table 2:** Summary of the remuneration of physicians in Israel

**Setting**	**Sub setting**	**Remuneration**
Primary care physicians	Clinic based	• A monthly salary, based on experience, list size and the number of hours worked
		An additional monthly capitation payment for patient lists that are longer than a prescribed number
		• Additional special payments for certain activities
	Independently based	• Capitation basis
Community based specialists	Salaried	• Salary reflecting the number of hours worked, experience and rank
		• Additional payments for seeing more “first-time” patients
	Independent	• Capitation basis
		• Additional fee-for-service payments for some procedures
Hospital based doctors		• Mainly salaried depending on responsibility and experience.
		• Additional money through:
		○ Private work in private hospitals or community settings- usually fee-for-service
		○ Some voluntary hospitals in Jerusalem allow private services in public hospitals by out of pocket, supplementary or commercial health insurance schemes and fee for service.
		○ Working for established health trusts out of hours
		○ Some accept illegal, under-the table payments

Source: Health System Review [6].

### Why did the strikes occur?

Whilst there were a number of factors contributing to the 2011 physician strikes, most of those interviewed attribute the principal cause to low wages. Many interviewees reported that, despite their many years of competitive and intensive training, doctors perceive themselves to be relatively underpaid in comparison to their equals in other countries and in other professions, even following a general rise in their salaries over the last decade. Furthermore, many Israeli physicians complete international fellowships in countries where doctors have higher salaries and a better standard of living, which becomes their reference point on return home. One interviewee reported that some doctors feel cheated when they see the comparison; that *‘they give so much to their country’s health system to get so little in return’*. Some Israeli doctors have supplemented their wages through private work and believe that if the government wants them to remain in the public sector, not only should they be paid more, but that they should see their pay increase as supplementary insurance uptake increases. There is also demand to allow private practice in public hospitals to facilitate higher earnings. The message from our interviews was that the Israeli government is however resistant to physicians’ demands for higher pay in the fear that it would encourage other public sector workers to also insist on major pay increases, feeling that it would not have been in a position to respond, especially following the 2008 financial crisis.

Whilst the current strikes may have been in part about pay, doctors in many countries are unhappy or seek higher payments yet do not strike [14]. Although some of these countries have laws prohibiting physician strikes, others do not and this strongly suggests that there are some specific features of the Israeli health system that require attention. However, we also note that, even where strikes are illegal, they sometimes happen. A more detailed legal analysis of a large number of countries, which would require contact with knowledgeable key informants in each, is outside the scope of the paper.

One of the slogans of the 2011 strikes was ‘save our public health system’ and this was certainly a key theme of the IMA campaign. Some physicians were concerned with the increasing privatisation of the health system, poor access in certain areas of the country, overcrowding in hospitals, a shortage of beds and staff (especially in the peripheries and certain specialities), poor working conditions and insufficient resources to deliver an effective health system, with a general decrease in government health spending over the years since the NHIL came into effect [1,15]. Some commentators argue that, while a real concern, the argument for saving the public health system was actually a public relations stunt by doctors and the IMA to make the real issue of higher wages more palatable to the public. Yet, our interviews suggest that there were certainly doctors who truly believed in it and saw it as their primary concern.

The context of the strikes is also important. Strikes are commonplace in Israel, considered a part of ‘Israeli mentality’ especially amongst the medical profession. According to four of those interviewed, some Israeli doctors believe that striking is the only way to gain attention and achieve their goals. During the resolution of the 2000 strikes, a ten-year no-strike agreement was made. As this period came to an end, physicians became increasingly frustrated that little had changed and, according to our interviewees, many doctors were just waiting and willing for the chance to strike again. Many doctors defended their right to strike whilst others would argue that it is never ethical to do so.

Other contributing factors to the strikes include the dual role of the IMA, which not only represents doctors but also sees itself as an advocate for the public and for the health system. Normally these dual roles of the IMA coincide, but not always. For example, in the recent negotiations, the IMA's push to increase pay for doctors in the remote parts of the country due to an interest in improving health in the periphery, may have prevented a larger pay increase for doctors in general. Some doctors therefore felt betrayed by the IMA leading to rifts within the association [1]. They saw the IMA as hiding behind a socialist flag, failing to prioritise their main role, which they saw as representing doctors, their salaries and pensions.

It is also important to consider that the 2011 strikes actually comprised two elements; an initial strike, following which the agreement was signed between the IMA and the Government, and a secondary wave of resignations by young doctors in the centre of the country who did not benefit significantly from the first agreement but who were unable to strike as this would have been illegal once the first strikes terminated. These younger residents felt unheard and unrepresented. They were encouraged by senior doctors working in the centre of the country, who also felt betrayed by the initial agreement, to resign due to the perception that most of the pay increases that followed the strikes went to doctors working in the periphery of the country, designed to encourage people to work there. Furthermore it should be noted that whilst the bonuses for work in the periphery or distressed specialties were very significant for those physicians who received the bonuses, overall they account for only a very small fraction of the total incremental cost of the new wage agreement see also [16]. Those threatening to resign had been hoping for a substantial bonus for full time public work or for legitimisation of private work in the public hospitals. According to some of those interviewed, those involved in this secondary wave appear to have been less concerned with the overall good of the public health system than the broader group of doctors involved in the first wave of strikes [17].

### Cracks within the health system?

In the previous section, we reviewed some of the possible reasons for the strikes. The key question that we are asking is whether the strike also reflects underlying problems with the health system. This was the view expressed by many of the protestors, many of whom directly attacked the health system, the government and Prime Minister Benjamin Netanyahu in particular who, unusually, combined this post with that of Health Minister. They chanted “Knesset (parliament), wake up, the health care system is crumbling [18]”. The IMA, in a position paper, explicitly framed the strikes as a mission to ‘improve the welfare of patients and protect the public healthcare system [4,19]’ while Leonid Eidelman, chair of the IMA said “the health system is collapsing [20]^”^. One leading academic commentator, Dov Chernichovsky, Chair of the Taub Centre Health Policy Programme, argued that “the strike reveals the depth of the structural crisis that has emerged within the healthcare system [21]^”^.

It is imperative however, to ensure that there is no confounding between slogans and the strikes' genuine objectives, as well as between the strikers' perceptions and the reality of Israeli health care. To assess whether the strikers' claims do amount to evidence of emerging cracks within the system we return to the WHO building blocks and the definition of the goals of a health system; to improve ‘*health and health equity in ways that are responsive, financially fair and make the best or most efficient use of available resources* ([10], p2)’. If there are emerging cracks, then the Israeli health system will not be achieving these goals, and the demands of the strikers should resonate with a fault within one or more of WHO’s six building blocks. Here we look at the six building blocks as the core structure of a health system, not to analyse systematically Israel’s health care system against each of them in their entirety, but to assess whether the strikers demands resonate with any of them individually.

### Faults in the health system building blocks as identified by the strikers

#### Health workforce

##### Shortage of healthcare professionals

There is increasing concern about a projected overall shortage of doctors, nurses and hospital beds as well as significant imbalances impacting adversely on remote areas and unpopular specialties [21] and this was a key message highlighted by those striking [22]. A shortage of doctors is something that few would have predicted a decade ago, when Israel was the destination for large numbers of doctors from Eastern Europe. However, the existing medical schools have not expanded to keep pace with the growing population and the supply of eastern European doctors has dried up. Reduced prestige of the medical profession in Israel, emigration of doctors, inadequate salaries, and burnout have also been cited as reasons for the projected shortage [22]. Other cited reasons include migration of the Israel public and physicians to the private system [1], internal ‘brain drain’ as well as a problem of geographic maldistribution of doctors rather than a doctor shortage *per se*. It should be noted, however that there is still no shortage of Israelis applying to medical schools, which are generally heavily oversubscribed, with entry remaining extremely competitive, and medicine remains one of the most popular courses of study when measured as the ratio of applicants to places [23]. The government is expanding training capacity, most notably with a new medical school that opened in Galilee in October 2011, but one interviewee noted that this will take some time to impact on the medical workforce and staff shortages are projected to *‘*get worse before they get better’*.* There is no specific body with responsibility for workforce projection and planning but *ad hoc* committees are set up to address this problem when needed [6].

**Figure 1:**
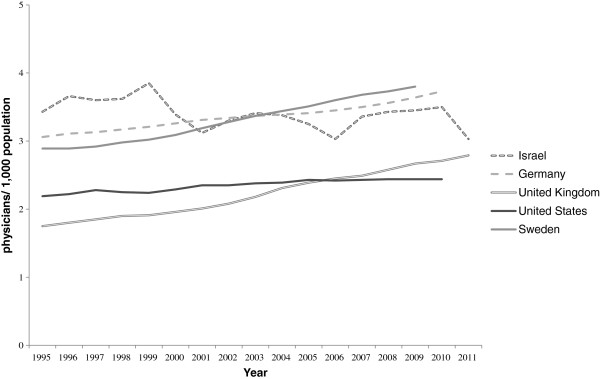
**Physicians per 1,000 population in selected countries.** Source: OECD health database [24].

It is, however, necessary to place the perceived shortage of doctors in an international context. The ratio of doctors to population is still higher than in many other countries (Figure 1). In 2010, it was estimated that the number of practising physicians for every thousand people was 3.5, although this figure fell to 3.0 in 2011 (with the sharp drop perhaps attributable to sampling errors). This compares with 2.4 in America and 3.1 in Organisation for Economic and Cooperation and Development (OECD) countries as a whole [1]. However, some other countries have overtaken Israel.

**Figure 2:**
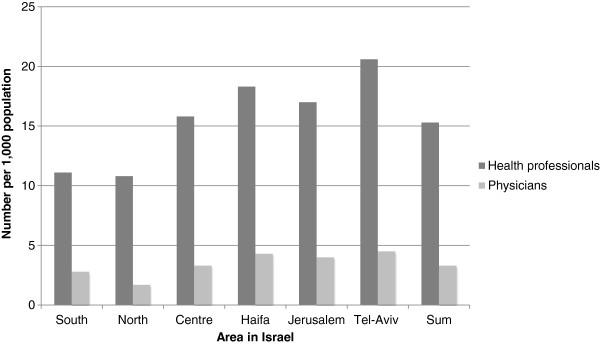
**Health professionals and physicians per 1,000 population.** Source: The Israel Ministry of Health [25].

It is also important to note that the number of health professionals per 1,000 population varies considerably according to region [1], with almost half as many health care professionals per 1,000 population in the periphery (North and South) compared with Tel-Aviv, as illustrated in Figure 2.

According to Chernicovsky and Regev [1] however, the ‘serious problem for which no reliable data exist is that of the siphoning of the physician manpower, particularly specialists, out of the public system and into the private market, and a consequent worsening of the relative shortage of physicians (especially specialists) in the public system’.

#### Workforce morale

Another concern is morale in the workforce; there is widespread agreement that this is low amongst Israeli doctors and that it has played a part in the genesis of the strikes. It is attributed to inadequate occupational pensions, long hours and what many perceive to be low pay, as discussed above. The OECD has compared the earnings of salaried doctors and nurses around the world and with all workers with tertiary education in twelve countries including Israel. They found that, in 2008, Israeli doctors and nurses had close to the average compensation of health professionals in other countries. In addition, Israeli specialists are paid over 2.2 times what university graduates earn in other sectors of the Israeli economy, with the figure for general practitioners about 1.4 times, both of which compare favourably with figures from other OECD countries [26].

Workforce morale deteriorated during the strike negotiations when it was proposed that doctors should ‘clock in and out’. From the government’s point of view this would prevent physicians from leaving the public settings in the early afternoon to pursue private work in private hospitals, a practice that has increased a great deal over the last 5–10 years, and would also allow doctors to be paid overtime. Many doctors felt it would undermine their professional integrity and, according to the chair of the IMA Ethics Bureau, would lead to “*a culture of doing only what you have to*” [20] thereby simply legitimising the behaviour of those leaving early to practice privately. For many doctors the ‘clock in clock out’ was an emotional issue, a ‘red flag’ that they felt undermined their professionalism leaving doctors ‘deeply insulted to the point of outrage by the doubts cast on their integrity’ [27].

Some doctors suggested a solution whereby doctors could practice privately in public hospitals thereby supplementing their income without cost to the government and allowing them to remain in the public setting. The government, however, made it clear that it was not going to negotiate private practice in public hospitals for two reasons: economically- if physicians practiced privately in public hospitals there would be increased demand, increased activity and increased health expenditure with no increase in the health level of the population; and on grounds of equity - the government wanted to prevent a two tier system of health that could also extend to other sectors such as education.

#### Service delivery

In general, the performance of the Israeli health system is accepted to be good [23]. For example, waiting times for community-based doctors are short overall, but there are some widely recognised concerns, such as inaccessibility, adequacy of medical explanations, cultural responsiveness and bedside manner that are problematic. There are also issues of inequality, overcrowding, an inability to keep up with changing health needs -for example as a consequence of an aging population- and insufficient hospital beds with too few medical personnel [1,6,15,21,28]. In 2010 the ratio of hospital beds per thousand people in Israel was 3.3 compared with an OECD average of 4.9 [24]. A key complaint by strikers was the insufficient time doctors in the community are allocated for each clinic consultation. During the latest strikes the IMA also tried, as outlined above, to solve the issues surrounding the poorer level of care in peripheral parts of the country and the low number of doctors working in some specialties [2,4,6,20]. Interestingly, despite the claims of system failure, as noted above, the strikers did not focus to any significant extent on some of the known problems with the Israeli health system, such as the exclusion of long-term, dental and mental health care from the health plans [6], that ‘*opportunities for engaging in screening and health promotion are quite limited*’ ([6] p198), and longstanding concerns about inequity, affecting in particular elderly people, immigrants, Israel’s Arab minority and the poor [6]. Notably, these issues did feature more prominently in previous strikes by Israeli doctors.

#### Financing

Many strikers saw the cause of the current problems as inadequate health care financing and to some extent, challenges to equity. Historically, financing of the Israeli health system has been relatively equitable, especially since the implementation in 1995 of the health insurance law, but this has been somewhat eroded by privatisation and commercialisation of aspects of the health system that is arguably moving towards an American system of care. In 2009, Israel spent 7.9% of its GDP on health, compared to the OECD average of 9.5%, with a total expenditure on health of US$2,071 per capita (expressed as purchasing power parity) in 2010, compared with an OECD average of US$2,754. Although this figure is slightly higher than in 1995 (7.4%), the share of public funding dropped from about 70% in 1995 to 60% in 2010, (the OECD average is 71.7%) [21,24] and, as is discussed above, about 80% of the public now have supplementary insurance. There are two views on this; one, that the share of public funding has decreased simply as a consequence of changing expectations leading to increased private spending, and the other, that the increased private spending has been necessary to compensate for decreased public spending, with increased co-payments and supplemental insurance [6,21]. As the private system strengthens, the public system gets weaker leading to a widening of inequalities and the threat of a two-tier system. As the Opposition leader at the time, Tzipi Livni, argued during the strikes, there are “two medical systems, one for the rich and one for the poor [20]”. Although people can, in theory, chose whether or not to purchase private insurance, the fact that 80% of people do so is a strong indicator that the system is failing to meet all of the public's wishes and perceived needs [6].

#### Leadership and governance

There are many different loci of leadership in the Israeli health system that include: the government as a whole, the Ministry of Health, the Ministry of Finance, hospital directors, health fund directors, heads of academic communities, and the IMA. Here we will focus on health leadership by the government. Leadership is about ‘overseeing and guiding the whole health system ([10] p23)’. Good leadership should take the health system forward. The NHIL is widely seen as having been a great achievement, but, since then, progress has been perceived by some to have stagnated [29] with some academics calling for ‘refreshment and policy amendments that will correspond to its original aspirations’ ([30], p273). Some believe that the Israeli health system lacks leadership, which is manifest in the examples of poor planning for staff shortages and unequal access documented above. For example, one member of the Knesset demanded that the Prime Minister “stop hiding behind representatives of the Treasury and Health Ministry and show real leadership [31]”. This may reflect frustration with a failure to reach agreement after eight months of unresolved negotiations.

Two other important factors preventing strong leadership are that the Ministry of Health is both a regulator, employer and provider of health services, leading to conflicts of interest [6] and that two ministries are involved with healthcare; both the Ministry of Health and the Ministry of Finance, which are not always on the same side. The multiple voices of the government complicate the situation, although it should be noted that these factors are not unique to the health ministry and are true of other social ministries. Crucially, the Ministry of Health is weaker than the Ministry of Finance, for several reasons; firstly, the structure of government in Israel that deliberately gives greater power to the Ministry of Finance; secondly, the Ministry of Health is both a regulator and provider of services; and finally, funding is controlled by the Ministry of Finance so the Health Ministry has to compete for finances in a country where health is still a relatively low priority [32,33]. Interestingly, when the NHIL was introduced, this was a rare time when the Ministry of Health, despite objections from the Ministry of Finance, was strong enough to implement a major health policy change.

There are however others, including some of those we interviewed, who argue that leadership of the Israeli health system is very good, perhaps even more proactive than it has ever been. Indeed there have been many major reforms recently, for example with respect to expansion of the benefits package and health equity; new reforms in the organisation of mental health services, the elimination of co-payments for mother and child and securing greater funds overall for the health system. Other changes include new legislation that makes it easier for those studying medicine abroad to get a licence to work in Israel and a major new initiative on health promotion and laws to limit smoking in public places.

How can these two views be reconciled? It could be that, in general, leadership of the Israeli health system is strong, but that the lack of leadership lies in managing and dealing with the physicians’ frustrations, particularly with their incomes, during each wave of strikes. In particular, one interviewer highlighted that a criticism coming out of the strikes is that the leadership did not listen to the residents who felt unheard and who should have been involved with negotiations from the beginning.

#### Information

Information includes the generation and dissemination of information about health system performance [10]. Whilst there have been many conferences and high level discussions on the strikes, regular news coverage, as well as closer analysis in the Hebrew literature, to an outside observer it is surprising how little in depth analysis there has been so far of the latest strikes in the academic or professional press. In particular, there has been little on the specific issues of physician incomes, their determinants and their implications. Many of those interviewed agreed that there was not as much as there could, or should have been. Similarly, although there is commentary in the mass media (in English and in Hebrew), there is very little published academic analysis of many of the previous strikes. Without critique and analysis, a system cannot learn.

Further analysis should not come only from a medical and health policy point of view but also from the perspective of sociologists and historians. One interviewee highlighted a number of broader social changes challenging the health workforce, including a change in work ethic, which some have attributed to an increased emphasis on work-life balance, affecting both genders, and this is something that others have noted [27]. Changing expectations may have contributed to the 2011 strikes, although this is something that should be explored in further research.

#### Medical products, vaccines and technologies

This is the only aspect not identified by the strikers during this wave of strikes as problematic, and is one of Israel’s strengths [6], although there is always some general frustrations as to what goes into the benefit package but this will always be difficult when prioritisation must occur. It has also been suggested that there is scope for technological developments to compensate for staff shortages [21].

### Outcome of the strikes

There were several positive outcomes from the strikes. These included an increase in government sponsored medical positions and increased resources and staff for the peripheral parts of the country. According to some interviewees, this improvement in health equity was one of the most successful outcomes of the strikes and something that had never been emphasised before. However, all interviewees agreed that many issues remain outstanding.

According to the IMA [34], only a year after the latest set of negotiations many doctors remain unhappy, even with an average salary increase of 12.1%. Doctors complain that there are still shortages of physicians in some specialties, shortages during weekend shifts and there is persistent disgruntlement about the new requirement of clocking in and out [34]. Furthermore, as we have already discussed, many of the doctors in the centre of the country felt left out of negotiations as most of the salary increases made at the end of the strike were for doctors in the periphery of the country, as an incentive for doctors to work there.

Disunity was also created between some of the senior and junior doctors with the more junior doctors feeling that the IMA had neglected their needs leading to division between the junior doctors and the IMA leadership [1]. However, as we saw above, some of the senior doctors had encouraged the resignations and threats of the junior doctors, to seek changes relevant to themselves rather than the residents, such as private practice rights and full-timer bonuses. Some doctors felt that the strikes were a battle between them and the Ministry of Finance and that ultimately the treasury had won on every account, humiliating the doctors and preventing them from further strikes for another ten years. According to one interviewee, the agreements following the strikes had actually made things worse. As a result of some of these problems, a new doctors’ organisation was set up called "Arbel", ‘consisting largely of younger doctors in the centre of the country’ or ‘renegade physicians that opposed the Israel Medical Organization’s policies in reaching an agreement with the Finance Ministry [35]’ causing further splintering amongst the profession. Another group involved was Mirsham (Medical Residents Working to Improve Israeli Medicine) which ‘sought to represent Israel’s younger resident physicians who it was claimed were paying the price of the public systems inadequacies [1]. Importantly, where doctors remain dissatisfied, there is a danger of further strikes.

Chernichovsky and Regev [1] argue that ‘it is highly doubtful whether the strike helped resolve any structural issues within the system, or whether it indeed saved Israeli public medicine, as the strike’s organizers originally intended….it appears mainly to have resolved certain point-specific issues within the system, rather than the fundamental problems: the declining share of public investment in the healthcare system, the lack of a long-term plan, and the private system’s growing share at the public system’s expense – a situation liable to worsen existing inequities and to impair the public system’s effectiveness’. This changing public-private mix of financing of the health system is a key problem with the health system that needs to be addressed, as highlighted recently by the Trachtenberg committee, a public committee for socio-economic change, appointed by Prime Minister Benjamin Netanyahu in August 2011.

## Conclusion

Contrary to the view held among some sections of Israeli society, strikes by physicians in the rest of the world are neither commonplace nor inevitable. Strikes should not be accepted as something that is simply part of Israeli medical culture; rather, the root causes should be carefully analysed and identified so that the system can move forward. The 2011 strikes were caused by a complex interaction of factors; it was largely due to dissatisfaction with salaries and working conditions, and disagreements regarding the public health system, but it was also in part a result of differences in interests, values and perceptions of reality between the Ministries of Health and Finance and the physicians. But do the strikes also reflect underlying problems within the health system?

It is important to remember that while most organised health systems offering universal coverage are hardly 100 years old [6], the introduction of the NHIL in 1995 makes Israel’s one of the newest; a teenager at 18 years. The NHIL was an achievement and has attained a high standard of care [6,28] that the majority of Israelis are happy with [21], the service is universal, there is an impressive basket of services, short waiting times and a high standard of medical training. In addition there is a good level of new technology and medications, the distribution of services is relatively equal, quality assurance mechanisms are in place [36,37], and satisfaction is generally high [15]. Furthermore, population health is generally good and the strikes were by no means a response to a health ‘crisis’.

However, if the goal of a health system is to improve ‘health equity in ways that are responsive, financially fair and make the best or most efficient use of available resources’ ([10] p2), then the analysis presented here suggests that strikes reflect cracks in the health system. The system is not as responsive as it might be; whilst it does deal with patients quickly when needed, the strikes still occurred despite eight months of negotiations. Although providing universal coverage, there is growing inequity, with co-payments and supplementary insurance playing a major role [38]. The system does not always make efficient use of resources as seen by staff shortages and limited hospital capacity. We have also seen, of the WHO’s six building blocks, weaknesses in five of them do resonate on some level with strikers demands. Furthermore, others argue that there are emerging cracks in the health system that were not even touched upon by the strikers. But what is the extent of these cracks?

Of the six WHO building blocks, three are most important in the present context. The first is ‘health workforce’. Dissatisfaction with salaries and conditions appears to be the main reason for the strikes; the workers feel that they are not listened to, and this is a complaint repeated with each set of strikes, including the latest strikes. Interestingly the 2000 and 2011 strikers made very similar demands [6] but these were not resolved in 2000 causing doctors to remain unsatisfied. This leads to the second key building block; leadership. This is most apparent in the inadequate management of physicians’ frustrations and complaints about their incomes. The third key building block is information. As this review has shown, while there are many opinions, there is a lack of in-depth rigorous analysis and critique of the strikes and their underlying causes. These three issues go hand-in-hand; whilst there is dissatisfaction amongst the workforce, only with careful understanding and analysis of the cause of the strikes can those at the top respond appropriately to these complaints and frustrations to lead the health system forward effectively.

As noted above, while strikes by physicians in other countries are rare, Israel is not unique. In this paper we have treated the Israeli strikes in the same way that hospital management might view a series of medical errors, as a critical event that, ideally, should not happen where a more detailed inquiry may reveal underlying systemic problems [39]. It seems reasonable to subject physician strikes in other countries to similar scrutiny. However, as with the Israeli strikes, only a very few published studies have done so [40,41], with most instead looking at issues such as the characteristics of strikers or the impact on the delivery of care or health outcomes [42-49].

In conclusion, while the strikes may be, in part, a reflection of cracks within the health system, many of these issues can be overcome. They should be seen as the teething problems of what is a relatively new, and still popular, health system that is still struggling with adolescence and looking for guidance as to where to go next.

## Abbreviations

IMA: Israel Medical Association; NHIL: National Health Insurance Law; OECD: Organisation for Economic and Cooperation and Development.

## Competing interests

The authors declare that they have no competing interests.

## Authors’ contributions

LW interviewed relevant sources and co-authored the paper. MM and GBN co-authored the paper. All authors read and approved the final manuscript.

## Authors’ information

LW is a doctor specialising in Public Health in the UK. She is completing a Masters in Public Health at the London School of Hygiene and Tropical Medicine. GBN is professor of Health Economics at the department of Health System Management, Faculty of management, Ben Gurion University of the Negev. MM is professor of European Public Health at the London School of Hygiene and Tropical Medicine.
